# Multimodal pressure-flow method to assess dynamics of cerebral autoregulation in stroke and hypertension

**DOI:** 10.1186/1475-925X-3-39

**Published:** 2004-10-25

**Authors:** Vera Novak, Albert CC Yang, Lukas Lepicovsky, Ary L Goldberger, Lewis A Lipsitz, Chung-Kang Peng

**Affiliations:** 1 Division of Gerontology, Beth Israel Deaconess Medical Center, Harvard Medical School, Boston, MA 02215, USA; 2 Margret and H. A. Rey Institute for Nonlinear Dynamics in Medicine and Cardiovascular Division, Beth Israel Deaconess Medical Center, Harvard Medical School, Boston, MA 02215, USA

**Keywords:** cerebral autoregulation, Hilbert-Huang transform, nonlinear dynamics, time-frequency analysis, stroke, Valsalva maneuver

## Abstract

**Background:**

This study evaluated the effects of stroke on regulation of cerebral blood flow in response to fluctuations in systemic blood pressure (BP). The autoregulatory dynamics are difficult to assess because of the nonstationarity and nonlinearity of the component signals.

**Methods:**

We studied 15 normotensive, 20 hypertensive and 15 minor stroke subjects (48.0 ± 1.3 years). BP and blood flow velocities (BFV) from middle cerebral arteries (MCA) were measured during the Valsalva maneuver (VM) using transcranial Doppler ultrasound.

**Results:**

A new technique, multimodal pressure-flow analysis (MMPF), was implemented to analyze these short, nonstationary signals. MMPF analysis decomposes complex BP and BFV signals into multiple empirical modes, representing their instantaneous frequency-amplitude modulation. The empirical mode corresponding to the VM BP profile was used to construct the continuous phase diagram and to identify the minimum and maximum values from the residual BP (BP_R_) and BFV (BFV_R_) signals. The BP-BFV phase shift was calculated as the difference between the phase corresponding to the BP_R _and BFV_R _minimum (maximum) values. BP-BFV phase shifts were significantly different between groups. In the normotensive group, the BFV_R _minimum and maximum preceded the BP_R _minimum and maximum, respectively, leading to large positive values of BP-BFV shifts.

**Conclusion:**

In the stroke and hypertensive groups, the resulting BP-BFV phase shift was significantly smaller compared to the normotensive group. A standard autoregulation index did not differentiate the groups. The MMPF method enables evaluation of autoregulatory dynamics based on instantaneous BP-BFV phase analysis. Regulation of BP-BFV dynamics is altered with hypertension and after stroke, rendering blood flow dependent on blood pressure.

## Background

Noninvasive assessment of cerebral vasoregulation is a major challenge in medical diagnostics and post-stroke care. Dynamic autoregulatory mechanisms adapt cerebral perfusion to spontaneous variations in intracranial and systemic pressure within a few heartbeats. Decline of cerebral blood flow that occurs with normal aging is further potentiated by presence of risk factors for cerebrovascular disease such as hypertension. Cerebral autoregulation is damaged by acute stroke, rendering cerebral blood flow dependent on blood pressure (BP) [1-3]. The duration of post-stroke autoregulatory impairment and the degree of recovery after stroke are not known. Stroke is more common in older subjects, but the consequences of stroke in younger subjects may last for decades. It is not known if cerebral autoregulation is impaired in subjects with minor chronic infarcts and good neurological outcome. This study, employing a new signal analysis method, addresses the unresolved issue of whether the dynamics of cerebral autoregulation are altered in younger subjects with minor chronic stroke.

Continuous monitoring of BFV using transcranial Doppler ultrasound enables assessment of dynamic autoregulation from spontaneous BP and BFV fluctuations [4], and during interventions inducing a sudden BP reduction, such as the VM, thigh cuff deflation, and the sit-to-stand test [5-7]. The VM induces a sudden increase in intrathoracic and cerebrospinal fluid pressure that is associated with rapid declines in BP and BFV. The BFV response to intracranial pressure changes precedes peripheral BP responses. The end of straining is followed by a BP increase ≈30 mm Hg above baseline, associated with an increase in BFV and cerebrovascular resistance [8,9]. BP and BFV fluctuations evoked by the VM are transient and highly nonstationary. Rapid changes in vascular tone that act to adjust perfusion pressure during these fluctuations may yield a nonlinear pressure/flow relationship. These short and nonstationary time series present a methodological challenge since they are not suitable for analysis using traditional analytic techniques based on Fourier transform analysis [4,10] or Volterra-Wiener moving average modeling [11] which assume signal linearity and stationarity.

Accordingly, we: 1) introduce a new method, multimodal pressure flow analysis (MMPF), based on the Hilbert-Huang transformation [12], to quantify the relationships between two nonstationary signals; 2) apply the MMPF method to the assessment of dynamic autoregulation using the instantaneous BP-BFV phase relationships during the VM; 3) compare pressure/flow dynamics in younger subjects with a minor chronic stroke to that of normotensive and hypertensive subjects without stroke, and 4) compare the MMPF method with standard indices of autoregulation in the stroke and non-stroke groups.

## Methods

### Subjects

Studies were conducted at the Autonomic Nervous System Laboratory at the Department of Neurology at The Ohio State University and at the SAFE (Syncope and Falls in the Elderly) Laboratory at the Beth Israel Deaconess Medical Center at Harvard Medical School. All subjects signed informed consent, approved by the Institutional Review Boards. Subjects in the stroke and non-stroke groups were recruited from the Neurology Stroke Service and through advertisement. Demographic characteristics are summarized in Table 1.

**Table 1:** Demographic characteristics and baseline blood pressure and blood flow velocities in MCAs

**Group**	**Normotensive**	**Hypertensive**	**Stroke**
**Men/Women**	9/6	8/12	5/10
**Age (yrs)**	40.2 ± 2.0	49.9 ± 2.0	53.1 ± 1.6**
**Race W/AA**	14/1	15/5	14/1
**Stroke side, R/L**	--	--	4/11
**Baseline values**			
**mean BP (mm Hg)**	84.2 ± 2.2	102.1 ± 3.0	97.2 ± 2.4***
**mean BFV MCAR (cm/s)**	53.1 ± 3.4	60.7 ± 3.7	59.5 ± 4.3
**mean BFV MCAL (cm/s)**	53.0 ± 4.3	58.5 ± 3.5	57.2 ± 3.8
**EtCO_2 _(mm Hg)**	37.5 ± 1.9	35.4 ± 1.3	36.2 ± 1.1

Demographic characteristics, age and baseline values (mean ± SE, ** p < 0.003, p < 0.0001), gender and race (W = white, AA = African American) and the stroke side (R/L = right/left), BP = mean blood pressure, BFV = mean blood flow velocity in the right (MCAR) and left (MCAL) middle cerebral artery, EtCO_2 _= end tidal carbon dioxide

#### Normotensive group

15 healthy normotensive subjects (age 40.2 ± 2.0 years, mean ± SE).

#### Hypertensive group

20 patients with essential hypertension, controlled by antihypertensive medications (age 49.9 ± 2.0 years).

#### Stroke group

15 subjects with the first minor ischemic stroke (18.3 ± 4.5 months after acute onset) (age 53.1 ± 1.6 years) including 9 subjects treated for hypertension (stroke-hypertensive) and 6 subjects who were normotensive (stroke-normotensive) with no BP treatment. Stroke subjects had a documented infarct on MRI or CT affecting <1/3 of the vascular territory with a minor neurological deficit (Modified Rankin Score scale <3). The side of the lesion was determined by neurological evaluation and confirmed with MRI and CT. The lesion was in the right hemisphere in 4 and in the left hemisphere in 11 subjects. The infarct types and locations were as follows: small vessel infarcts in 13 subjects and large vessel infarcts in 2 subjects. Cortical infarcts were found in 10 subjects and subcortical infarcts in 5 subjects. Normal carotid Doppler ultrasound study was required for participation. Patients with hemorrhagic strokes, clinically important cardiac disease including major arrhythmias, diabetes and any other systemic illness were excluded. All subjects were carefully screened with a medical history, physical and laboratory examination. Subjects with hypertension (with or without stroke) were treated with antihypertensive agents from the following categories (diuretics, beta-adrenergic blockers and angiotensin-converting enzyme inhibitors). Antihypertensive medications were gradually tapered over 3 days and discontinued for 3 days prior to the study. Anticoagulants and other medications that did not affect cardiovascular or autonomic nervous system function were allowed.

### Experimental protocol

#### VM

After instructions and several practice sessions, each subject rested for 5 minutes in the supine position. The subject was then asked to take a breath and expire forcefully through a mouthpiece with a small air-leak, maintaining for 15 seconds a pressure of 40 mm Hg monitored on a pressure gauge connected to the mouthpiece. All data were continuously acquired over the 5 minute period during which BP returned to baseline. The VM was repeated twice and the signal showing the more prominent VM oscillation by visual inspection was selected.

### Data acquisition, processing and analysis

The experiments were done in the morning or > 2 hours after the last meal. The electrocardiogram was measured from a modified standard lead II or III using a Spacelab Monitor (SpaceLab Medical Inc., Issaquah, WA). Beat-to-beat BP was recorded from a finger with a Finapres device (Ohmeda Monitoring Systems, Englewood CO), which is based on a photoplethysmographic volume clamp method. During the study protocol, BP was verified by arterial tonometry. With finger position at the heart level and temperature kept constant, the Finapres device can reliably track intraarterial BP changes over prolonged periods of time [13]. Respiratory waveforms were measured with a nasal thermistor. CO_2 _was measured from a mask using an infrared end tidal volume CO_2 _monitor (Datex Ohmeda, Madison WI). The right and left MCAs were insonated from the temporal windows, by placing the 2-MHz probe in the temporal area above the zygomatic arch using a transcranial Doppler ultrasonography system (MultiDop X4, DWL Neuroscan Inc, Sterling, VA). Each probe was positioned to record the maximal BFV and fixed at a desired angle using a three-dimensional positioning system attached to the light-metal probe holder. Special attention was given to stabilize the probes, since their steady position is crucial for reliable, continuous BFV recordings. BFV and all cardiovascular analog signals were continuously acquired at 200 Hz and exported at 50 Hz for off-line post-processing. Data were visually inspected and occasional extrasystoles and outlier data points were removed using linear interpolation. Fourier transform of the Doppler shift (the difference between the frequency of the emitted signal and its echo (frequency of reflected signal) was used to calculate BFV. BFVs in the MCA correlate with invasive measurements of blood flow with xenon clearance [14], laser Doppler flux [15] and positron emission tomography [16]. Since MCA diameter is relatively constant under physiological conditions, BFV can be used for blood flow estimates [17].

### Multimodal pressure-flow method

To quantify the dependency between cerebral blood flow and systemic pressure, we developed a novel computational procedure, called multimodal pressure-flow (MMPF) analysis. The MMPF analysis implemented the Hilbert-Huang transformation [12] technique to measure the coupling between two nonstationary signals. This method was motivated by the fact that the original BP and BFV signals are recorded over time. However, different subjects vary in the amount of time spent in each stage of the VM. Therefore, it is essential to find an alternative coordinate system for both BP and BFV signals that allows for a meaningful, non-time dependent cross referencing of these two signals. Since the complete VM cycle can be treated as a full cycle of BP oscillation, the oscillatory phase of the BP modulation during the VM can serve as such a useful coordinate system.

**Figure 1:**
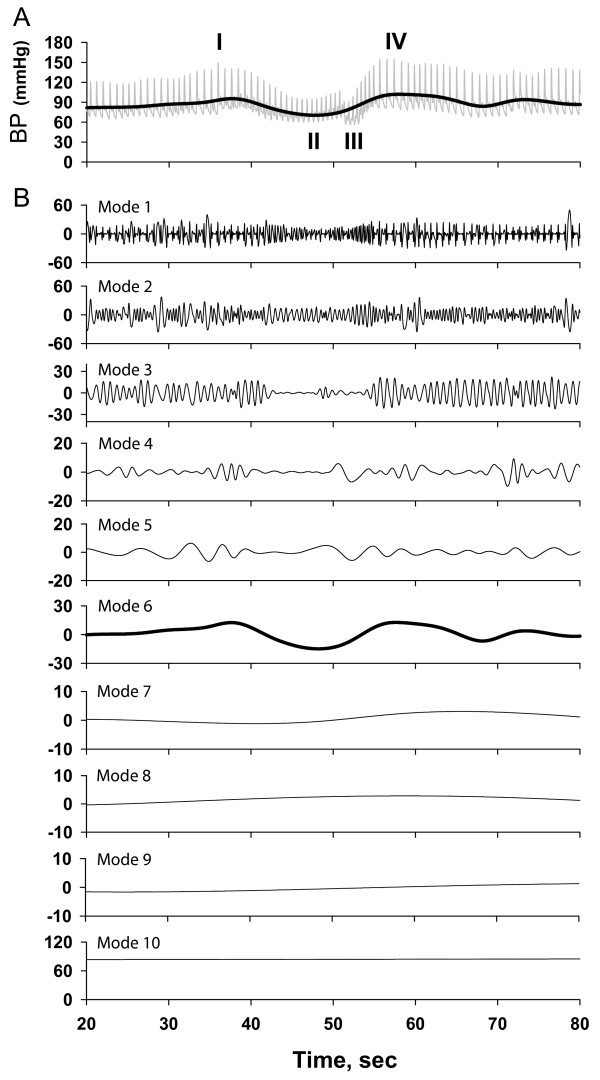
Schematic diagram showing Hilbert-Huang decomposition of the original blood pressure (BP) signal into the empirical modes corresponding to amplitude-frequency modulation for different time scales. Panel A shows the profile of the BP waveform over the course of the VM: I- indicates the beginning of the maneuver, II- the duration of straining, III-the end of straining and IV- the BP overshoot above baseline. (Note that the transient BP decrease in phase III is due to inspiration.) Panel B shows the empirical modes for each component frequencies and their corresponding amplitudes were detected from the signal (mode 1–10). Empirical modes corresponding to the faster frequencies (modes 1–5) were removed from the original BP and BFV signals. The empirical mode corresponding to the characteristic BP profile induced by the VM (BP_VM _– mode 6 in this example) was used to obtain phase information. Modes 7–10 reflect BP modulations at slow frequencies. Similarly, the empirical mode corresponding to the characteristic BFV profile was extracted from the raw BFV waveform (not shown).

To implement this approach, we first need to calculate how BP phase changes as a function of time. Then we can map the original time-varying BP and BFV signals to the new axis of reference, namely BP oscillatory phase. To precisely calculate the BP phase, we need to extract the characteristic (dominant) BP oscillation induced by the VM. We applied the empirical mode decomposition (EMD) technique developed by Huang et al. [12] The EMD algorithm decomposes complex signals such as BP and BFV into multiple empirical modes. Each mode represents the frequency-amplitude modulation at a specific time scale. Figure 1A shows the original BP waveform, which is modulated by multiple frequencies corresponding to the systolic peak, dicrotic notch, heart rate, respiratory frequency and BP fluctuations induced by the VM. Figure 1B shows the decomposed empirical modes (1–10) of the original BP signal. Empirical modes 1–5, corresponding to the faster frequencies, were removed from the signal. The remaining lower frequency BP signal, termed the "residual BP" or BP_R _(shown as thick curve in Figure 1A), was used to identify the maximum and minimum values during the VM. The empirical mode best representing the dominant BP profile during the VM, denoted as BP_VM _(mode 6 in this example, plotted as the thick line in Figure 1B), was visually identified and used for subsequent phase analysis. We followed the same procedure to obtain the residual BFV signal, denoted as BFV_R_.

In the next step of the MMPF analysis, we applied the Hilbert transform to the BP_VM _signal to calculate its instantaneous phases. This phase was then used as a reference coordinate both for BP and BFV signals. From this point on, the term *phase *refers to the phase of the BP_VM _oscillation during the VM. Unlike the Fourier transform, the Hilbert transform does not assume that signals are composed of superimposed sinusoidal oscillations of constant amplitude and frequency. Real-world biological fluctuations, such as BP and BFV, are not stationary and are better described by analytical methods that can quantify variations of amplitude and frequency.

Mathematically, the first two steps of the MMPF algorithm can be summarized in the following way: Any complex signal s(t) can be represented as the superimposition of more basic (simpler) components: *S*(*t*) = ∑_*k *_*S*_*k *_(*t*), where *S*_*k *_are empirical modes that fulfill certain criteria of the original signal [12]. For each empirical mode, its Hilbert transform is defined as:



where the Cauchy principal value is taken in the integral. Instantaneous amplitude, *A*_*k *_(*t*), and instantaneous phase, *φ*_*k *_(*t*), can be calculated by



The BP_VM _profile from the first peak at the beginning of the VM to the subsequent BP_VM _maximum forms a complete phase cycle from 0–360° over 30–40 seconds. We assigned the first peak at the beginning of the VM to phase 0°, the BP_VM _minimum during the VM to phase 180° and the subsequent BP_VM _maximum to phase 360°. To quantify the relationships between BP and BFV signals, we measured the BP-BFV phase shift, defined as the difference between the phase at the BP_R _minimum (maximum) value and the phase at the BFV_R _minimum (maximum) value. We have calculated phase values for all data points in this interval and, in principle, we can measure the phase shift at all point of the VM cycle. However, for simplicity, we only calculated the phase shift at the minimum and maximum of these two signals for statistical analysis. Since these BP-BFV phase shifts reflect dynamical changes in peripheral and cerebral vascular tone over the course of the VM, they can be used as a sensitive index of cerebral autoregulation dynamics in normal and pathological conditions.

### Autoregulation indices

We also assessed autoregulation using a standard index, calculated using the second-order differential equation model proposed by Tiecks et al[5] This model assumes a linear flow-pressure relationship and a constant cerebral perfusion pressure over the course of a sudden BP reduction, such as occurs during thigh cuff deflation. This technique can be also applied to the sudden BP decline during the VM. The autoregulation index ranges from 0 = "no autoregulation" to 9 = "the fastest autoregulation;" value 5 reflects "normal autoregulation." We also calculated the "rate of autoregulation" (RoR) using the slope of the linear regression fitted to the original BP and BFV waveforms signals during the period between the baseline and the BP minimum (descending slope) and between BP minimum and maximum (ascending slope).

### Statistical analysis

We used one-way analysis of variance for between-group comparisons of baseline, minimum and maximum BP_R _and BFV_R _values and phases. Two-way analysis of variance was used for side-to-side comparisons of BFV between groups (JMP-5.0 SAS Institute, Cary, NC). For the group comparisons, we used the BFV_R _in the right and left MCAs for the normotensive and hypertensive groups compared to the BFV_R _in the stroke-side and non-stroke side MCA in the stroke group. Age was different between the groups (p < 0.003). However, age and stroke subtypes had no significant effects when included as co-variants in the analysis. Data are presented as mean ± SE.

## Results

**Figure 2:**
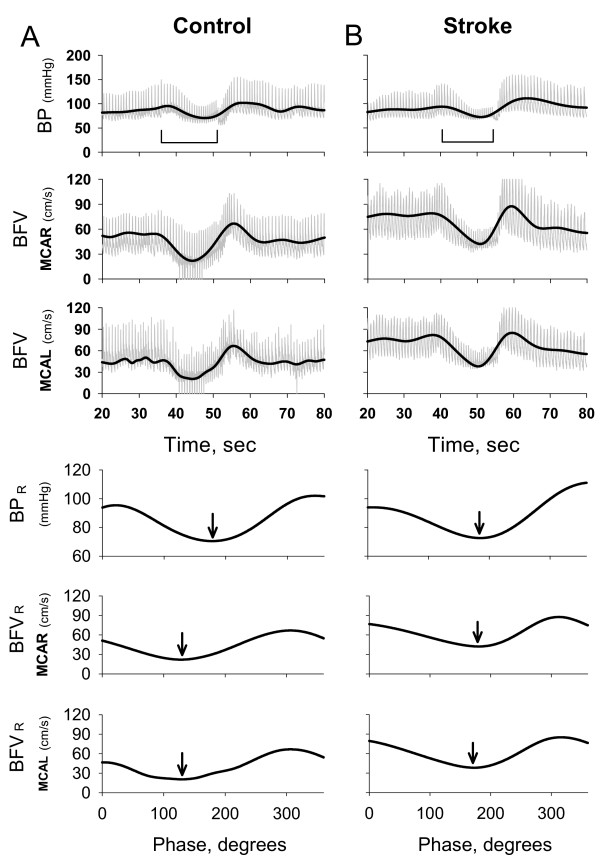
Panel A shows blood pressure (BP) and blood flow velocity (BFV) waveforms from the right and left MCAs (MCAR and MCAL respectively) during the VM for a normotensive subject (top 3 panels). The duration of the VM straining is indicated by a horizontal line. The thick black line indicates the BP_R _and BFV_R _that reflect the characteristic VM oscillation. Bottom 3 panels show BP_R _and BFV_R _in the MCAR and MCAL. Arrows indicate phases at the BP_R _and BFV_R _minima. With normal autoregulation, BFV_R _minimum preceded BP_R _minimum. Panel B shows BP and BFV waveforms for a subject with a left temporal infarct (MCAR = non stroke-side MCA, MCAL = stroke side MCA) (top 3 panels). Horizontal line indicates duration of the VM. Black thick line indicates the BP_R _and BFV_R _obtained from the BP and BFV raw waveforms. Bottom 3 panels show BP_R _and BFV_R _in the non-stroke side MCA and in the stroke-side MCA expressed as a function of BP_VM _phase. Arrows indicate that the phase at BFV minimum was similar to the phase at BP minimum.

Figure 2A shows representative raw BP and BFV waveforms (right and left MCAs) during the VM for a normotensive 31 year old man. The BP_R _and BFV_R _signals are superimposed on the raw waveforms. For comparison, we show similar signals (the right, non-stroke side, MCA and the left, stroke side, MCA) for a 48 year old woman with the left temporal stroke in Figure 2B. The BP_R _and BFV_R _for the right (non-stroke side) MCA and for the left (stroke side) MCA are shown in the bottom three panels as a function of the phase. With normal autoregulation, the BFV_R _changes precede BP_R _changes over the course of the VM. Therefore, the phase at the BFV_R _minimum is smaller than the phase at the BP_R _minimum. (Phase at the BFV_R _minimum for the right MCA = 129° and the left MCA = 112° vs. the phase at BP_R _minimum = 178°.) In contrast, with post-stroke cerebral autoregulation, the phase at the BFV_R _minimum is similar to the phase at the BP_R _minimum, suggesting that BFV is dependent on blood pressure. (Phase at the BFV_R _minimum for the non-stroke MCA = 173° and for the stroke MCA = 180° vs. the phase at BP_R _minimum = 185°).

### Pressure flow relationship at the BFV_R _minimum

**Figure 3:**
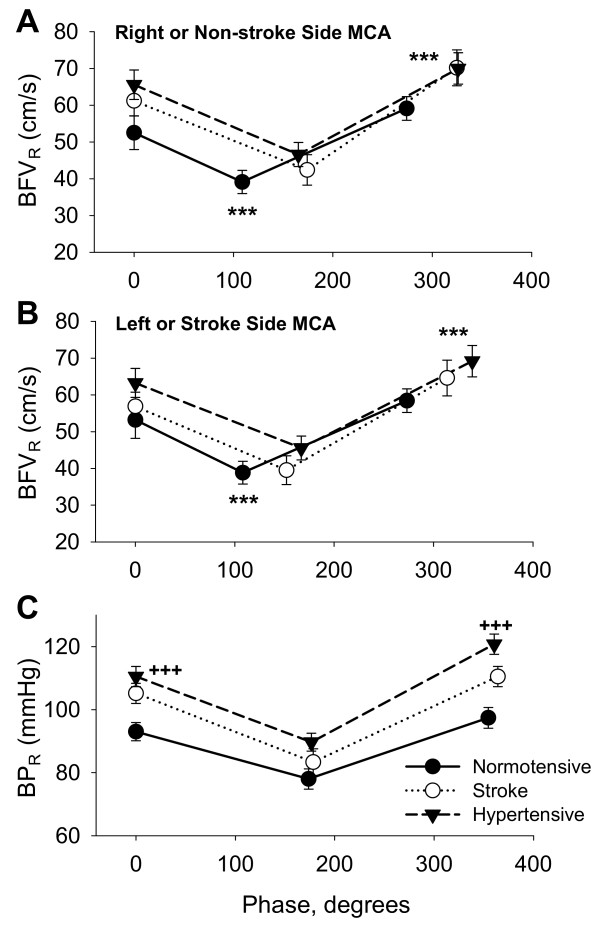
Panel A shows the phase and corresponding residual blood flow velocity (BFV_R_) values at baseline, BFV_R _minimum and BFV_R _maximum for the right MCA for the normotensive -●- and - -▼- hypertensive groups and for - O- the non-stroke side MCA in the stroke group. Panel B shows the phase and corresponding BFV_R _values for the left MCA in the normotensive and hypertensive groups and for the stroke side MCA in the stroke group. BFV_R _phase was significantly greater in the stroke and hypertensive groups compared to the normotensive group for BFV_R _minimum and maximum in both MCAs (between groups phase comparisons *** p < 0.005, ** p < 0.01). Panel C shows the phase and corresponding residual blood pressure (BP_R_) values for the BP_R _minimum and maximum (between groups BP_R _values comparisons +++ p < 0.001, mean ± SE).

Figure 3A shows the group averages of BFV_R _values and the phases at the BFV_R _minimum and maximum values for the right MCA in the normotensive and hypertensive groups and for the non-stroke side MCA in the stroke group. Figure 3B shows the BFV_R _values and the phases for the left MCA in the normotensive and hypertensive group, and the stroke side MCA in the stroke group. Mean BP_R _values and corresponding phases are shown in panel C. The phase at BFV_R _minimum was different between groups for the right (non-stroke side) (p = 0.0005) and left (stroke side) (p = 0.004) MCAs. In the normotensive group, the phase at BFV_R _minimum was shorter than the phase at BP_R _minimum. In the stroke group, the phase at BFV_R _minimum was similar to the phase at BP_R _minimum. The phase at BFV_R _minimum was greater in the non-stroke (p = 0.002) and stroke (p = 0.03) MCAs, compared to the normotensive group. In the hypertensive group, the phase at BFV_R _minimum was also greater for the right (p = 0.0007) and left (p = 0.006) MCAs compared to the normotensive group. No significant differences were found between the stroke and hypertensive groups.

The phase at BP_R _minimum was similar between groups (normotensive group = 172.7 ± 3.9°, hypertensive group = 178.6 ± 2.0°, stroke group = 178 ± 1.9°). Average BP_R _values were higher in the stroke and hypertensive groups compared to the normotensive group at baseline (p = 0.001), at BP_R _minimum (p = 0.054) and at BP_R _maximum (p = 0.0001) (Figure 3C). Average BFV_R _values at baseline, at BFV_R _minimum and maximum, and BFV_R _change from baseline were not different among groups. Average BP_R _change and percent change from baseline to BP_R _minimum and maximum were not different. Average BFV_R _change and percent change from baseline to BFV minimum and maximum were also not different.

### Pressure flow relationship at the BFV_R _maximum

In the normotensive group, the phase at BFV_R _maximum preceded the phase at BP_R _maximum (Figure 3). The phase at BFV_R _maximum was different between groups for the right (non-stroke side) (p = 0.009) and left (stroke side) (p = 0.003) MCAs. In the stroke group, the phase at BFV_R _maximum was similar to the phase at BP_R _maximum. The phase was greater for the non-stroke side (p = 0.008) and stroke side (p = 0.03) MCAs, compared to the normotensive group. In the hypertensive group, the phase at BFV_R _maximum was also greater for the right (p = 0.005) and left (p = 0.009) MCAs compared to the normotensive group. The phase at BP_R _maximum was similar among groups (normotensive group = 354 ± 4.7°, hypertensive group = 360.1 ± 2.2° stroke group = 364.1 ± 4.3°).

### BP-BFV phase shifts

**Figure 4:**
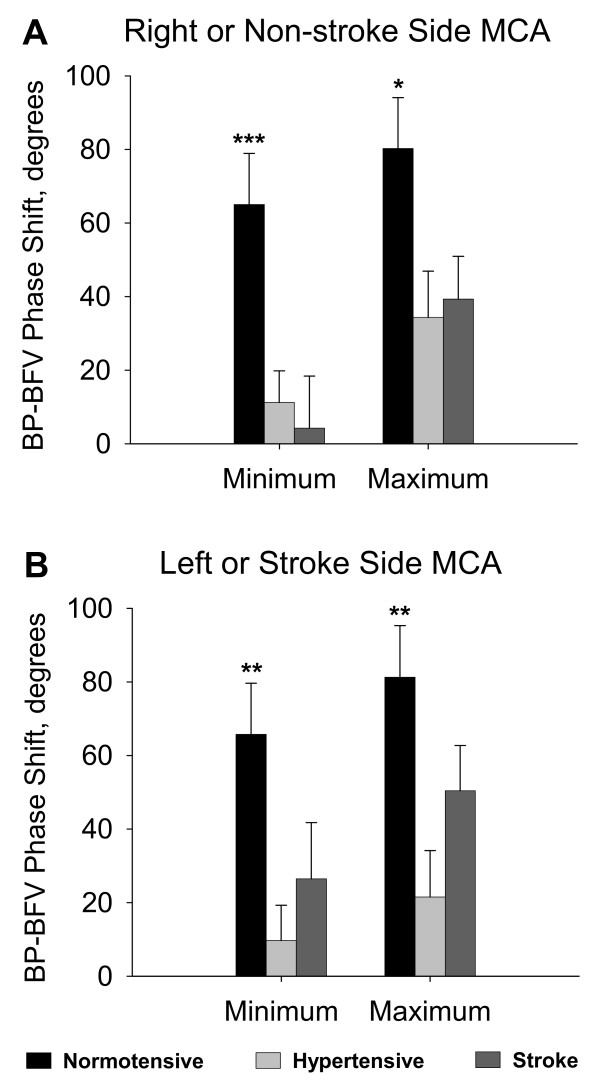
Panel A shows the phase shift between BP_R _minimum and BFV_R _minimum and the phase shift between BP_R _maximum and BFV_R _maximum for the right MCA for the □ normotensive and hypertensive groups and ■ for the non-stroke side MCA in the stroke group. Figure 4B shows the phase shift between BP_R _minimum and BFV_R _minimum and the phase shift between BP_R _maximum and BFV_R _maximum for the left MCA for the normotensive, and hypertensive groups and for the stroke side MCA in the stroke group. Phase shift was greater in the normotensive compared to other groups (between group comparisons *** p < 0.005, ** p < 0.01 *p < 0.05, mean ± SE).

Figure 4A summarizes the differences between the phases at BP_R _and BFV_R _minimum and between the phases at BP_R _and BFV_R _maximum for the right MCA in normotensive and hypertensive groups and for the non-stroke side MCA in the stroke group. The BP-BFV phase shifts at the minimum points were smaller in the stroke and hypertensive groups compared to the normotensive group (p = 0.009). The BP-BFV phase shifts at the maximum points were smaller in stroke and hypertensive groups compared to the normotensive group (p = 0.03). Figure 4B shows the phase shift for the left MCA in normotensive and hypertensive groups and for the stroke-side MCA in the stroke group. The BP-BFV phase shifts at the minima and maxima were also smaller in the stroke and hypertensive groups compared to the normotensive group (p = 0.0002). The BP-BFV phase shifts at the maxima were smaller in the stroke and hypertensive groups compared to the normotensive group (p = 0.008). In the stroke group, the BP-BFV phase shift at the maxima was greater compared to the phase shift at the minima for the non-stroke MCA (p = 0.02). The BP-BFV phase shifts at the minima and maxima for the stroke MCA did not reach statistical significance (p = 0.08). The BP-BFV phase shifts between the stroke and non-stroke MCA at the minima and maxima were not different. In the normotensive group, the phase difference between BP_R _and BFV_R _minima was about 60 degrees corresponding to a time difference of about 3.6 seconds. In contrast, in the stroke and hypertensive groups, the time difference between BFV and BP phases was < 0.5 second.

### Autoregulation indices

The standard autoregulation index was not different between groups and between MCAs in both hemispheres (Table 2). The rate of autoregulation (RoR) of BFV responses to BP reduction and increases during the VM were also not different (Table 2).

**Table 2:** Autoregulation Indices

**Variable**	**Normotensive**	**Hypertensive**	**Stroke**
**MCA side**	Right	Right	Non-stroke-side
**ARI**	5.7 ± 3.3	6.1 ± 2.6	6.1 ± 2.6
**RoR – descending slope**	0.7 ± 0.1	1.1 ± 0.6	0.6 ± 0.2
**RoR – ascending slope**	0.5 ± 0.1	1.6 ± 1.0	0.6 ± 0.2
**MCA side**	Left	Left	Stroke-side
**ARI**	5.9 ± 3.2	5.9 ± 2.6	5.1 ± 1.9
**RoR-descending slope**	0.6 ± 0.1	0.8 ± 0.4	0.5 ± 0.2
**RoR-ascending slope**	0.5 ± 0.1	1.5 ± 1.0	0.6 ± 0.2

Autoregulation index (ARI) and the rate of autoregulation (RoR) for the right and left MCAs in the normotensive and hypertensive groups and for the non-stroke and stroke side MCA in the stroke group. RoR – descending slope of the linear portion of the BP and BFV reduction between the baseline and BP minimum. RoR – ascending slope of the linear portion of the BP and BFV increase between BP minimum and maximum during the VM. ARI and RoR were not significantly different between the groups. Data are presented as mean ± SE

### Stroke-normotensive and stroke-hypertensive subjects

In a subset analysis, we separated stroke-normotensive (N = 6) and stroke-hypertensive subjects (N = 9) and compared them to the non-stroke normotensive and hypertensive groups. For the non-stroke side MCA, the phases at BFV_R _minima (p = 0.01) and maxima (p = 0.04) were greater in the stroke-normotensive group compared to the normotensive group. For the stroke side MCA, the phases at BFV_R _minima (p = 0.04) and maxima (NS, p = 0.08) were greater in the stroke-normotensive group compared to the normotensive group. The phases at BFV_R _minima and maxima were not different between the stroke-hypertensive and hypertensive groups for both MCAs. No significant differences were found between the stroke and non-stroke side MCA.

## Discussion

This study introduces a new technique, based on the Hilbert-Huang transformation [12,18], termed multimodal pressure-flow analysis, for assessing the relationships between systemic blood pressure and cerebral blood flow changes associated with provocative maneuvers. Development of this method was motivated by the facts that 1) that the duration of the VM stages and resulting BP and BFV responses vary over time and among subjects, and 2) these types of time series are short and nonstationary, and therefore, not suitable for analysis using standard Fourier transform and autoregressive type approaches. We implemented the MMPF method to evaluate the dynamics of cerebral autoregulation using the instantaneous systemic BP and MCA BFV phase relationships during the VM. The frequency and corresponding BP_R _and BFV_R _amplitudes were computed for each data sample to construct a continuous phase diagram. The BP_R _and BFV_R _profiles were similar over the course of the VM, but the phase relationships were different. The autoregulation indices, calculated using the standard methods, did not differentiate the groups.

Cerebral vasoregulation compensates for rapid BP and BFV transitions over the course of the VM. A sudden and parallel increase in intrathoracic [19] and cerebrospinal fluid [8] pressures is associated with a rapid decline in BP and an initial increase of cerebrovascular resistance, resulting in a rapid decline in BFV. With active autoregulation, cerebrovascular resistance diminishes, enabling BFV to recover in the face of falling perfusion pressure, and BFV responses in the MCA precede the systemic BP changes. Therefore, in healthy controls, the phases corresponding to the BFV_R _minimum and maximum were smaller than BP_R _phases, reflecting an active vasoregulatory process. With delayed or impaired autoregulation, BFV becomes synchronized with blood pressure. In the stroke group, the BFV_R _and BP_R _phase diagrams were similar, suggesting that cerebral blood flow was entrained by systemic BP. In the stroke and hypertensive groups, BFV phases at the BFV_R _minimum and maximum were greater compared to the normotensive group. The BP-BFV phase shifts were smaller in the stroke and hypertensive group, compared to healthy controls.

Methods evaluating dynamic cerebral autoregulation that use the Fourier transform-based coherence and transfer functions assume a linear relationship between stationary signals. Coherence, phase and gain derived from the transfer function of spontaneous BP and BFV fluctuations have been used to assess autoregulation. These analyses have shown a significant phase lead of cerebral BFV with respect to systemic BP [4,20,21]. However, assumptions about signal stationarity and a linear flow/pressure relationship are not met for the short nonstationary time series from the VM, and therefore transfer function gain was not evaluated. The joint time-frequency distributions [22], such as the new MMPF developed here, that make no assumptions about signal characteristics and can reliably track simultaneous changes of spectral powers and frequencies, are better suited for these short nonstationary signals. The variable time delay between BP and BFV suggests that the relationship between the cerebral blood flow and systemic pressure is not linear.

Previous studies indicated bilateral impairment of the dynamics of pressure autoregulation after an acute [2,3] and subacute ischemic stroke [23]. Dynamic indices of autoregulation that were calculated from spontaneous BP and BFV fluctuations were altered, with no significant difference between autoregulatory indices in the affected and unaffected hemispheres. No significant differences were found in autoregulatory indices between large vessel anterior and posterior circulation infarcts and lacunar infarcts [2]. In patients with large hemispheric strokes, head elevations from 0° to 45° induced reductions in arterial BP, BFV, intracranial and perfusion pressures [1]. We have reported [24] that cerebral vasomotor responses to hypocapnia and hypercapnia were diminished following minor chronic infarctions. Baseline BFVs in the MCAs were similar between the stroke and the non-stroke groups, but differed during head-up tilt. BFV declined in the stroke side MCA during the head-up tilt. Side-to-side BFV differences were the most prominent in stroke-normotensive subjects with lower BP during head-up tilt compared to stroke-hypertensive subjects and non-stroke groups. The present study has confirmed bilateral impairment of autoregulation dynamics in stroke-normotensive and stroke-hypertensive subjects and also in a non-stroke hypertensive group. The BP-BFV phase relationships suggest that the autoregulatory responses were delayed in both hemispheres and that the BFV responses were dependent on perfusion pressure. About one third of stroke patients are hypertensive upon hospital admission. Hypertension and stroke may exert similar pathophysiological effects on vascular compliance, sympatho-vagal interactions [25] and blood pressure regulation [26,27]. Increased vascular stiffness, impaired vasodilatation and shift of the autoregulatory responses toward higher BP values may also affect the timing of autoregulatory responses in both hemispheres.

There are several limitations of this study: 1) The MMPF method was implemented for the VM, which is widely used for clinical autonomic testing. The VM allows noninvasive evaluation of pressure autoregulation, and testing can be completed in less than 5 minutes. However, the VM requires active patient cooperation, and may not be advisable in acute stroke settings where change in intracranial pressure should be avoided. 2) This study evaluated a population of younger subjects with minor stroke. A larger cohort is needed to determine the effects of ischemic stroke subtypes on the dynamics of autoregulation. 3) Age was different between groups; however, it had no significant effect on BP-BFV relationship in our analysis. Aging and cardiovascular risk factors exert significant but distinct effects on regulation of cerebral blood flow. The vasomotor reactivity to hypercarbia declines with aging and hypertension, while the dynamics of pressure regulation can be preserved [7]. This effect may be in part due to a shift of the autoregulatory range toward higher blood pressure values.

## Conclusions

Multimodal pressure-flow analysis is a new method that enables evaluation of short nonstationary time-series not suitable for Fourier–based techniques. The MMPF method provides high time and frequency resolution and permits construction of instantaneous phase diagrams on a beat-to-beat basis. This method may be particularly useful as a complementary measure of cerebral autoregulation for the short and nonstationary time series acquired during provocative interventions such as the VM. Application of this method reveals that the regulation of BP-BFV dynamics is altered in both hemispheres after minor stroke, rendering blood flow dependent on blood pressure. Hypertension without stroke is also associated with delayed BP-BFV dynamics.

## List of abbreviations

BP = blood pressure

BP_R _= residual BP calculated by MMPF method

BP_VM _= empirical mode of BP corresponding to the dominant VM oscillation

BFV = blood flow velocity in the MCA

BFV_R _= residual BFV calculated by MMPF method

EMD = empirical mode decomposition

HHT = Hilbert-Huang Transform

MCA = middle cerebral artery

MMPF = multimodal pressure-flow analysis

VM = Valsalva maneuver

## Competing interests

The author(s) declare that they have no competing interests.

## Authors' contributions

VN – designed the study, conducted the experiments, participated in the analysis and wrote the first draft of the manuscript. ACY – contributed to the MMPF development and analysis; LL – conducted the data and statistical analysis. ALG – contributed to the method application, data interpretation and manuscript preparation. LAL – contributed to data interpretation and manuscript preparation. CKP – designed and developed MMPF method, contributed to data analysis and manuscript preparation.
